# Developing a preoperative predictive model for ureteral length for ureteral stent insertion

**DOI:** 10.1186/s12894-016-0189-8

**Published:** 2016-11-30

**Authors:** Takashi Kawahara, Kentaro Sakamaki, Hiroki Ito, Shinnosuke Kuroda, Hideyuki Terao, Kazuhide Makiyama, Hiroji Uemura, Masahiro Yao, Hiroshi Miyamoto, Junichi Matsuzaki

**Affiliations:** 1 Department of Urology, Yokohama City University, Graduate School of Medicine, 3-9 Fukuura, Kanazawa-ku, Yokohama, Kanagawa 2360004 Japan; 2 Departments of Biostatistics, Yokohama City University Graduate School of Medicine, Yokohama, Japan; 3 Department of Urology, Ohguchi Higashi General Hospital, Yokohama, Japan; 4 Departments of Pathology and Urology, Johns Hopkins University School of Medicine, Baltimore, USA

**Keywords:** Ureteral length, Ureteral catheter, Predictive model, Ureter, Ureteral stent

## Abstract

**Background:**

Ureteral stenting has been a fundamental part of various urological procedures. Selecting a ureteral stent of optimal length is important for decreasing the incidence of stent migration and complications. The aim of the present study was to develop and internally validate a model for predicting the ureteral length for ureteral stent insertion.

**Methods:**

This study included a total of 127 patients whose ureters had previously been assessed by both intravenous urography (IVU) and CT scan. The actual ureteral length was determined by direct measurement using a 5-Fr ureteral catheter. Multiple linear regression analysis with backward selection was used to model the relationship between the factors analyzed and actual ureteral length. Bootstrapping was used to internally validate the predictive model.

**Results:**

Patients all of whom had stone disease included 76 men (59.8%) and 51 women (40.2%), with the median and mean (± SD) ages of 60 and 58.7 (±14.2) years. In these patients, 53 (41.7%) right and 74 (58.3%) left ureters were analyzed. The median and mean (± SD) actual ureteral lengths were 24.0 and 23.3 (±2.0) cm, respectively. Using the bootstrap methods for internal validation, the correlation coefficient (R2) was 0.57 ± 0.07.

**Conclusion:**

We have developed a predictive model, for the first time, which predicts ureteral length using the following five preoperative characteristics: age, side, sex, IVU measurement, and CT calculation. This predictive model can be used to reliably predict ureteral length based on clinical and radiological factors and may thus be a useful tool to help determining the optimal length of ureteral stent.

## Background

Since the first description by Zimskind et al. in 1967 [1], ureteral stenting has been a fundamental part of various urological procedures for the treatment of obstructing ureteral calculi, ureteral stricture, ureteropelvic junction obstruction, retroperitoneal tumor and fibrosis, and the procedures that were developed after open or endoscopic ureteral surgery [2]. Placement of a stent that is too long often causes complications, such as frequent or urgent urination, incontinence, hematuria, and bladder or flank pain, which have a negative impact on quality of life of patients [3–9]. Conversely, a short ureteral stent increases the risk of migration, resulting in complications that require retraction and replacement [10, 11]. Thus, choosing a stent of optimal length is important for reducing the incidence of stent migration and other complications [10, 12–14]. In our previous study, 4% of the ureteral stents were found to be too short and 19.5% were too long [15].

Actual ureteral stent measurement is the most accurate method to measure the ureteral length. However, actual stent measurement requires additional radiation exposure around 5.2 s and operation time of 0.2 min [15]. Moreover, in some hospitals, assorted lengths of ureteral stents are not stocked. Therefore, preoperative prediction of ureteral length is often needed. We previously reported the usefulness of direct measurement with a ureteral catheter in predicting the actual ureteral length [15]. In another study, we showed the reliability of multiple modalities for approximating the ureteral length in the same group of patients [16]. The length from renal vein to ureteral orifice measured by CT scan (axial CT distance: ACTD) showed a stronger association than any other variables tested, including body height (BH) and intravenous urography (IVU) measurement [17]. Accurately predicting the ureteral length is necessary for determining the optimal stent length [10, 12].

The aim of the present study was to develop and internally validate a preoperative predictive model for predicting the actual ureteral length (AUL).

## Methods

### Patients

We measured the ureteral length in 362 patients by direct measurement, as described below, at Ohguchi Higashi General Hospital (Yokohama, Japan) from 2010 to 2014. In these patients, a total of 127 ureters had previously been assessed by both IVU and CT scan, preoperatively. This study was approved by the Review Board of Kanagawa Prefecture Medical Association (Approved number: H25-3169) and written informed consent was obtained from the patients for their data to be used for research purpose.

### Measurement of actual ureteral length

The AUL, which was defined as the length between the ureteropelvic junction (UPJ; detected by fluoroscopy after retrograde pyelography) and the ureteral orifice (detected by cystoscopy), was determined by direct measurement using a 5-Fr ureteral catheter (TigerTail®, BARD, Murray Hill, NJ, USA), as we previously described [15]. In order to avoid overly straightening of the ureter, which might decrease its length, we measured the AUL, using a ureteral catheter without the guidewire.

### Preoperative factors

The correlations between the actual ureteral length and the following variables were assessed: (1) BH, (2) age, (3) sex, (4) side, (5) IVUa (the linear distance from the UPJ to the uretero-vesicle junction [UVJ] by IVU), (6) IVUb (the linear distance from the mid-kidney to the UVJ by IVU), (7) IVUc (the ureteral trace was measured by tracing the ureter from the ureteropelvic junction to the ureterovesical junction and measuring the length of the trace with a flexible ruler which was in the form of a string by IVU), and (8) the distance from the level of the renal vein to the ureteral trace by non-contrast ACTD. ACTD was the distance from the renal vein level to the urinary orifice level. It was defined as the number of CT slices multiplied by the distance between each slice using axial CT. The upper slice was defined as the level of the renal vein, and the lower slice was defined as the level of UVJ.

### Statistical analysis

Variables were selected by backward selection. Multiple linear regression analysis with backward selection was used to model the relationship between the factors analyzed and actual ureteral length. The selection criterion of *p* < 0.1 was used for elimination of a variable. Some variables were initially excluded from the model because of multicollinearity. Before multiple linear regression, we exclude IVUc, because of the strong association between IVUa and IVUc (r = 0.924). Decisions with respect to the coding of the predictive model variables were made before variable selection. A bootstrapping method was a nonparametric data generating method in which new datasets were repeatedly generated from an original dataset. Using the bootstrap methods for internal validation, the coefficient of determination (variance explained, R2) was assessed. Statistical analyses were performed using SAS 9.3 (SAS Institute Inc., Cary, NC, USA).

## Results

**Fig. 1:**
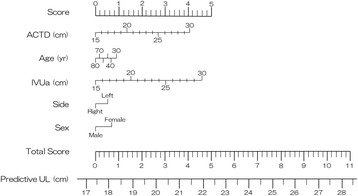
Predictive model predicting ureteral length

A total of 127 patients all with stone disease, including 76 men (59.8%) and 51 women (40.2%), were enrolled in this study. The median and mean (± SD) ages were 60 and 58.7 (±14.2) years (range: 28 - 86), respectively (Table 1). Fifty-three (41.7%) were right ureters (41.7%) and 74 (58.3%) were left ureters (58.3%). The median and mean (± SD) AULs were 24.0 and 23.3 (±2.0) cm (range: 16.0 – 27.5). Left side ureter are significantly longer than right side (23.7 ± 1.69 versus 22.7 ± 2.2, *P* = 0.007) Ureteral length in male was not longer than in female (23.4 ± 1.86 versus 23.1 ± 2.15, *P* = 0.350). On the other hand, multiple linear regression showed that sex was an independent factor (Table 2). The results of multiple linear regression are presented in Table 2. This predictive model contained five characteristics, including age, side, sex, IVUa, and ACTD. The total score of the predictive model was derived from the sum of the individual scores of each predictive variable (Fig. 1). The predicting ureteral length was written as follow formula; Ureteral Length = 10.1 + 0.27 x ACTD (cm) - 0.016 x Age + 0.302 x IVUa (cm) + 0.504 x side (Right:0, Left:1) + 0.716 x Sex (Male:0, Female:1). Using the bootstrap method, the explained variance (R2) was 0.57 ± 0.07 (range; 0.291 to 0.805).

**Table 1:** Patients’ characteristics

Variables	Number (%) or median (mean ± SD)	Range (min., max.)
Ureteral Length (cm)	24 (23.3 ± 2.0)	16.0 - 27.5
Age	60 (58.7 ± 14.2)	28 - 86
Side		
Right	53 (41.7%)	
Left	74 (58.3%)	
Sex		
Male	76 (59.8%)	
Female	51 (40.2%)	
Body Height (cm)	163 (161.7 ± 9.7)	128 - 180
IVUa (cm)	24.0 (23.9 ± 2.8)	14.0 - 30.0
IVUb (cm)	26.0 (25.7 ± 2.7)	15.0 - 32.0
IVUc (cm)	20.5 (24.8 ± 2.7)	15.0 - 31.0
ACTD (cm)	22.4 (22.9 ± 2.7)	16.1 - 22.4

*IVU* intravenous urography, *ACTD* axial CT distance

**Table 2:** Multiplelinear regression for ureteral length (cm)

Variables	Coefficients	Standard error	*P* value
Intercept	10.130	1.580	<0.0001
Age	-0.016	0.009	0.010
Sex	0.716	0.259	0.007
Side	0.504	0.247	0.043
IVUa	0.302	0.054	<0.0001
ACTD	0.027	0.005	<0.0001

## Discussion

We previously reported the effectiveness of loop-type ureteral stent that decreased the prevalence of stent-related symptoms, compared with double pigtail ureteral stents. Significantly lower scores for almost all of stent-related symptoms, other than nocturia, were seen with loop-type ureteral stents than in double pigtail stents [18]. On the other hand, even when using with loop-type ureteral stent, the stent-related symptoms were not prevented with overlong ureteral stent position (unpublished data). We previously demonstrated the relationship between the AUL and the ureteral stent position using direct ureteral length measurements in 226 patients with loop-type ureteral stents [15]. The migration rate and overlong rate were correlated with the ureteral length, when the proximal end of the stent was in the renal pelvis. The appropriate length of ureteral stent was the same or up to 1 cm shorter than the measured ureteral length. In that study 89.0% of loop-type ureteral stents with the same length of the ureter were appropriately positioned, whereas 11.0% seemed to be overlong than AUL. When loop-type ureteral stents were 1 cm shorter than AUL were placed, 88.6% were in appropriate positions, 8.6% were too long, and 2.9% had migrated into the ureter. In addition, loop-type ureteral stents that were more than 2 cm shorter than the measured ureteral length increased the incidence of migration to more than 10% [15]. Direct measurement with a ureteral catheter is thought to be the optimal method for selecting the appropriate length of ureteral stent, but the patient and operator are exposed to additional radiation and an extra procedure is required. Thus, while the direct measurement of the ureteral length is ideal, this predictive model may represent a new, more accurate method for estimating the ureteral length.

It is easy to apply the BH in predicting the ureteral length. Various studies have shown the association between BH and the ureteral length or the ureteral stent position [3, 16, 19, 20]. However, Shah et al. demonstrated that they were not significantly associated [21]. Gregory et al. reported the association of some variables, including body habitus, with ureteral lengths based on Vitruvius’ and da Vinci’s theories of proportion [22]. The AUL was significantly correlated with the BH, followed by the distances from the xyphoid process to the umbilicus (X-P) and from the shoulder to wrist (S-W), but they concluded that it was difficult to predict the ureteral length.

We previously investigated to define the best modality for estimating ureteral length in patients undergoing ureteral stent placement [17]. ACTD showed the strongest correlation (R^2^ =0.381) with AUL, compared with IVUa (R^2^ = 0.274), IVUb (R^2^ = 0.230), IVUc (R^2^ = 0.206), BH (R^2^ = 0.098), and body surface area (R^2^ = 0.095). However, when inserting loop-type ureteral stent, more accurate methods for predicting ureteral length are needed because of easy to migrate to the ureter, because the differences of 1 cm might change the risk of migration and increase the ureteral stent related symptoms.

In the current study, we developed a predictive model using the preoperative factors identified by multiple regression analysis: age, side, sex, length measured by IVU, and ACTD. Interestingly, IVUc which is a trace of the whole ureteral length (according to the curve of ureter) did not show a stronger correlation with the AUL. We hypothesize that when we measured the AUL, the ureteral catheter straightened the ureter, causing the AUL to shorten. We chose IVUa because it was most highly correlated with the AUL and because it was strongly correlated with each of the IVU methods. To our knowledge, this is the first report of a predictive model that predicts ureteral length. This predictive model showed the coefficient of determination (R^2^ = 0.566).

There are limitations in the present study. The endpoint of this predictive model was ureteral length, but not stent position or ureteral stent-related symptoms. We are now conducting a study to assess the stent position, using this predictive model. Furthermore, when a validated ureteral stent symptom questionnaire (USSQ) becomes available in Japan, we plan to conduct another study. The second limitation is that this predictive model is for patients who received both CT and IVU, not CT or IVU only. We previously showed the formula for estimating the actual ureteral length; however, this predictive model was found to have greater accuracy [17]. The third limitation is that we did not assess the background characteristics of the patients, including whether they were pregnant or had a history of radiation treatment, which might have influenced the length of the ureter. In this study, we only obtained data from patients who were treated for stone disease. We therefore speculate that these patients did not have a significant effect on the predictive model. On the other hand, this predictive model is not suitable for use with pregnant patients or patients undergoing radiation therapy. The forth one is that the same data was used for both variable selection and parameter estimation there is a risk that overfitting has occurred. Until an external validation is conducted model fit estimates are like to be inflated.

## Conclusion

We have developed a predictive model, for the first time, which can precisely predict ureteral length, using the five preoperative clinical and radiographical factors. This predictive model may thus be a useful tool to help determine the optimal length of a ureteral stent.

## Abbreviation

ACTD: Actual CT distance
AUL: Actual ureteral length
IVU: Intravenous urography
UPJ: Uretero pelvic junction
UVJ: Uretero vesicle junction
